# Nanobodies in animal infectious disease control: diagnosis and therapy

**DOI:** 10.3389/fcimb.2025.1640352

**Published:** 2025-07-25

**Authors:** Jing Wang, Tiejin Tong, Qiang Wu

**Affiliations:** School of Advanced Agricultural Sciences, Yibin Vocational and Technical College, Yibin, China

**Keywords:** nanobodies, animal infectious diseases, diagnostics, therapeutics, phage display, PRRSV, ASFV

## Abstract

Animal infectious diseases threaten livestock productivity, public health, and food security. Traditional monoclonal antibodies (mAbs) face limitations in diagnostics and therapy due to their large size, instability, and high cost. Nanobodies (Nbs), derived from camelid heavy-chain antibodies, offer superior properties—small size (~15 kDa), high stability, deep tissue penetration, and cost-effective production. Nbs feature extended CDR3 loops, enabling access to cryptic epitopes, and exhibit exceptional thermal/pH stability. They are generated by immunizing camelids, cloning VHH genes, and screening via phage/yeast display. High-throughput methods (ELISA, flow cytometry) allow rapid isolation of high-affinity Nbs. Compared to mAbs, Nbs are economically produced in prokaryotic systems and engineered into multivalent or Fc-fused formats for enhanced efficacy. In diagnostics, Nbs enable sensitive, low-cost detection of pathogens like PRRSV, ASFV, and avian influenza. Nb-based competitive ELISAs and lateral flow assays improve field surveillance. Therapeutically, Nbs neutralize pathogens by targeting viral proteins (e.g., blocking PRRSV-CD163 entry) or bacterial toxins (e.g., Staphylococcus enterotoxins). Nb-Fc fusions degrade ASFV proteins via TRIM-away, while intracellular Nbs disrupt Mycobacterium ESAT-6 or Toxoplasma actin dynamics. Challenges remain in Nb affinity optimization, intracellular delivery, and *in vivo* half-life. Solutions include fusion with cell-penetrating peptides or viral vectors (e.g., adenoviruses). Reducing cross-species immunogenicity and scaling production are critical for broader adoption. With advances in protein engineering, Nbs hold transformative potential for preventing, diagnosing, and treating animal diseases, offering scalable solutions for global health and food security.

## 1 Introduction

Animal infectious diseases are major obstacles to the healthy development of the livestock industry, posing serious threats to human health and public health security, particularly with the increasing occurrence of emerging and re-emerging diseases in recent years (Torres-Velez et al., 2019; Chala and Hamde, 2021). These emerging diseases are often characterized by sudden and large-scale outbreaks due to delayed implementation of control measures or the lack of effective diagnostic tools and vaccines, resulting in substantial economic losses to the livestock sector and productivity (Pathakumari et al., 2020). As the ideal approach for preventing and controlling animal infectious diseases, vaccination is still the most effective way, yet, limited by techniques, no effective vaccines have been prepared for most animal diseases (Bezbaruah et al., 2022). Hence rapid, sensitive, and specific diagnosis becomes particularly important in the prevention and control of animal diseases. High specificity and sensitivity of antibodies serve as reagents most suitable for developing diagnostic sera, and provide great potential for therapeutic applications. Therefore, antibody plays a key role in the prevention and control of animal infectious diseases (Thiviyanathan and Gorenstein, 2012). The antibodies development has passed through three stages: polyclonal antibody, monoclonal antibody, and small-molecule antibody (nanobody represented antibody) (Lipman et al., 2005; Thiviyanathan and Gorenstein, 2012; Medina Perez et al., 2024). The antibody used in practice first time is polyclonal antibody, because the specificity of the antibody is relative low, it is not very useful. In order to obtain antibodies which possess higher specificity, monoclonal antibodies were developed (Ramdani et al., 2022). These antibodies can bind to specific antigens and showed tremendous promise in various clinical uses such as alleviating viral infections and cancers. With the development of genetic engineering tools, researchers have further engineered antibodies to achieve better functional properties (Nema and Nitika, 2023). Because of the complicated nature of the traditional monoclonal antibodies, relatively large molecular weight and expensive production cost, some limitations exist when monoclonal antibodies are used in practice. Different with the traditional monoclonal antibodies, small-molecule antibodies (nanobodies, Nbs) is small with a simple structure and more convenient to modify genetically, which are more conducive to large scale production and application (Jin et al., 2023).

## 2 Overview of Nbs

**Figure 1:**
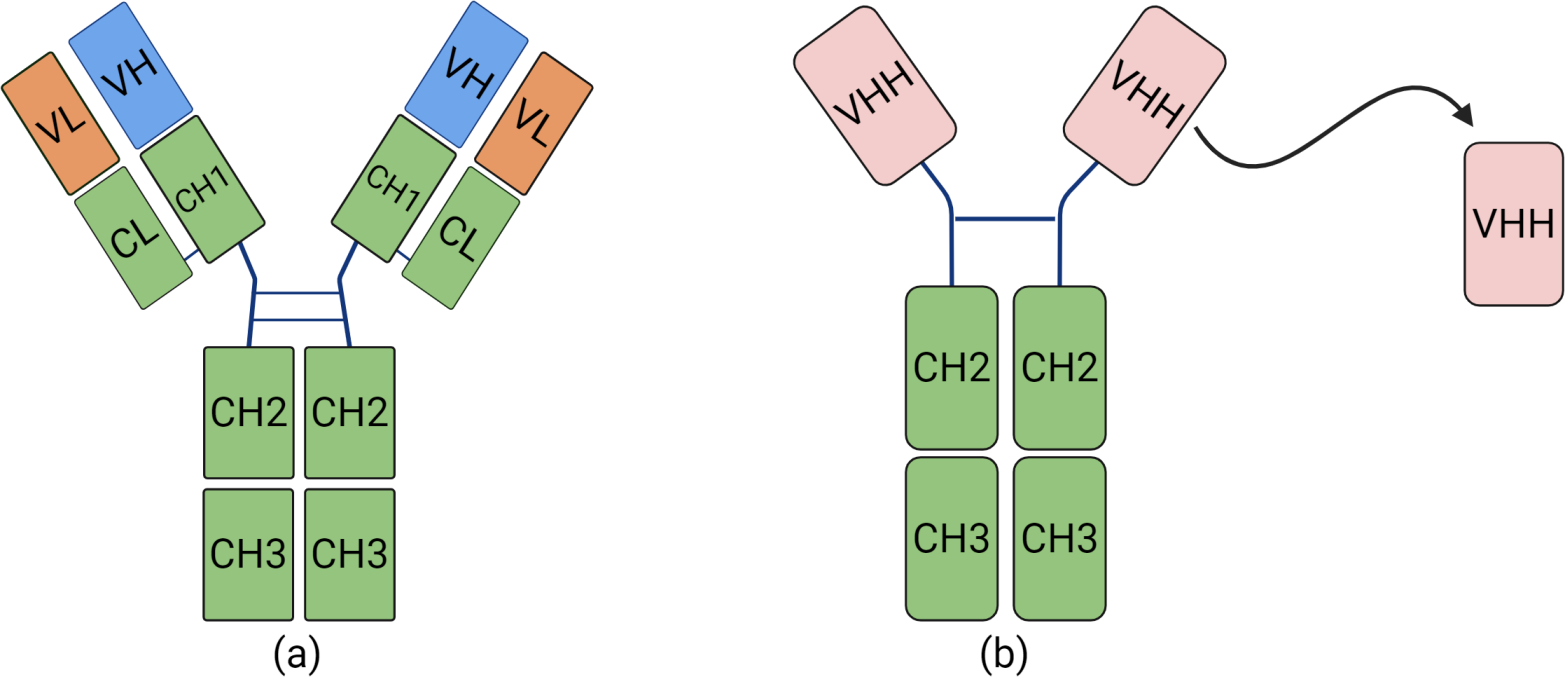
The differences between traditional monoclonal antibodies and Nbs. **(A)** Structural diagram of a conventional antibody, consisting of heavy and light chains. **(B)** Structural diagram of a nanobody.

Nbs are natural heavy chain antibodies (HCAbs) devoid of their light chains. This novel type of antibodies was originally identified and christened by a team at the University of Brussels in 1989. HCAbs are present in members of the Camelidae family and are distinguished in that they do not feature the CH1 domain that serves as a pairing partner of light chains (Muyldermans, 2013). Rather, HCAbs are formed by two heavy chains each comprised of a single variable antigen-binding domain (VHH) (Qin et al., 2022) (**Figure 1**).

HCAbs can bind to almost any kind of antigen and are the smallest naturally occurring, stable antigen-binding units, and thus named “nanobody”. Nbs are small ellipsoidal proteins, that structurally comprise a crystallizable Fc region, which is homologous to other antibodies (Percipalle et al., 2021). They have potential to operate as monomeric antibodies binding to a wide variety of antigens. In comparison with traditional antibodies, Nbs present various benefits:

Small Size:

Nbs are the smallest antibody fragments identified to date, at about one tenth of the size of normal antibodies. Nbs are defined by a longer-than-expected complementarity-determining region 3 (CDR3), responsible for the antigen-binding site. Because Nbs have a very small size and a particular shape, they can access an epitope, usually located deep in folded proteins (clefts, pockets, grooves, exposed surface). Notably, small size doesn’t hamper their binding affinity (Liu et al., 2023).

Favorable Biochemical Properties:

Besides the size benefits, there are other biochemical benefits associated with Nbs. They are very stable even at high-temperature stress, a feature attributed to long CDR3 sequences and ability to re-fold after denaturation. Thermal stability can be further improved with the choice of Nbs featuring an extra disulfide bridge, extending the CDR3 area or replacement of certain amino acid residues at the N-terminus. In addition, Nbs are highly soluble. The hydrophilic framework region 2 (FR2) prevents them from aggregating and enables them to behave as monomers. They are furthermore insensitive towards proteolytic activity and changes in pH, and on account of their microsize tissue penetration is promoted (Mujic-Delic et al., 2014).

Ease of Production:

Nbs can be conveniently expressed in conventional prokaryotic expression system or in mammalian eukaryotic expression system. If they are fused with affinity tag like a His-tag, they can be readily purified by immobilized metal affinity chromatography (IMAC). Alternatively, if Nbs are fused with Fc domains that are homologous to human VH regions, they can be purified by protein A affinity chromatography (Gonzalez-Sapienza et al., 2017).

## 3 Generation of Nbs

**Figure 2:**
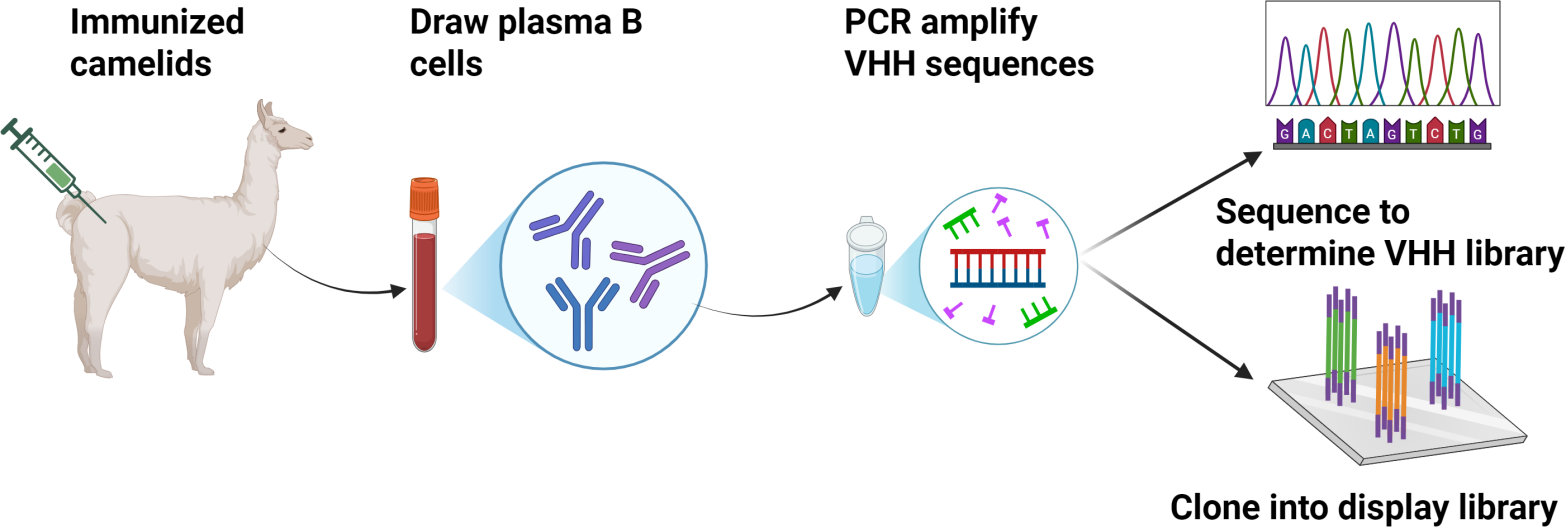
The preparation process of Nbs.

Owing to their incredible small size, Nbs have a number of advantages in comparison to monoclonal antibodies (mAb) and single-chain variable fragments (scFv), such as excellent affinity to unveil hidden epitopes, exceptional stability at low pH and high temperatures, genetic easiness in engineering, the ability of being expressed in prokaryotic hosts and low production costs (Jin et al., 2023). Due to these, Nbs are a promising alternative to generate antibody-based therapy for medical use. To elicit an antigen-specific Nb response, camelid animal (typically a llama or a camel) can be immunized with the target antigen (Jovcevska and Muyldermans, 2020). The acquired Nb sequences are then further synthesized into a phage or yeast display system to form an immune library and subjected to downstream screening/selection. The standard procedure for the selection of antigen-specific Nbs consists of the following steps: Camelids (e.g., alpacas) are immunized repeatedly over 3–10 weeks with the target antigen. Peripheral blood mononuclear cells (PBMCs) are then harvested, total RNA is isolated, complementary DNA (cDNA) is generated with random primers and after a nested PCR, using primers specific for Nbs, the VHHs are amplified. The amplified VHH fragments are inserted to an appropriate expression vector and the recombinant plasmids are transformed into *Escherichia coli* or yeast cell for expression. High-affinity Nbs candidates are selected by highthroughput methods as enzyme-linked immunosorbent assay (ELISA), or display technologies based on flow cytometry (Ding et al., 2023). The screening enables the qualitative selection of Nbs showing a high binding to the target antigen (**Figure 2**).

Immunize llamas with target antigens, isolate peripheral blood lymphocytes, extract RNA to construct VHH phage libraries via PCR amplification, screen for antigen-specific Nbs, then express and purify the selected Nbs for diagnostic or therapeutic applications.

## 4 Nb screening technologies

**Figure 3:**
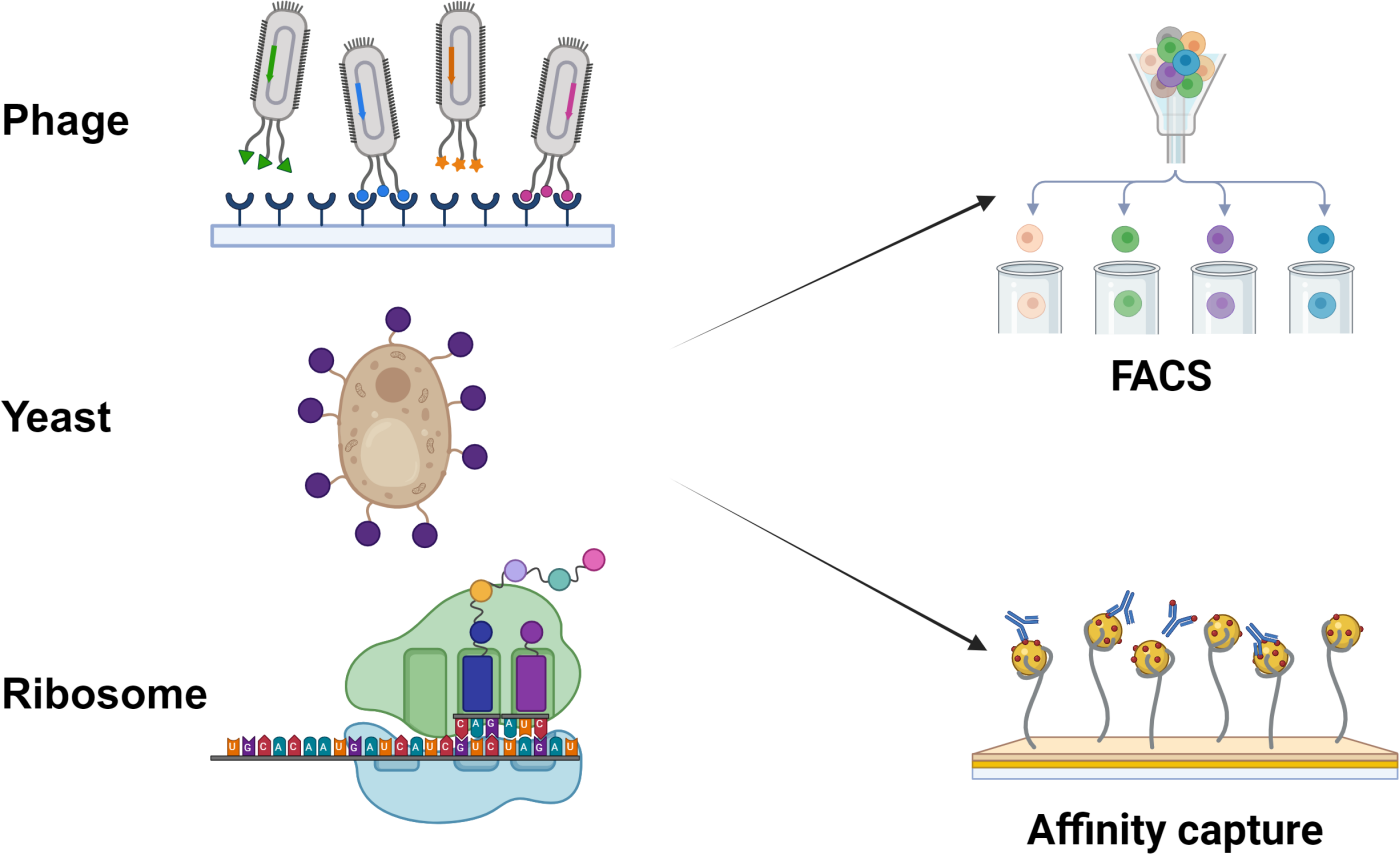
Nb screens different surface display platforms.

Surface display platforms that permit Nb screening are of different types such as phage display, yeast surface display, bacterial surface display, ribosome display and eukaryotic cell display systems (**Figure 3**). These can be integrated with high throughput identification systems like flow cytometry and next-generation sequencing (NGS) systems, and can be used with computer-assisted affinity maturation and *in vitro* optimization systems to optimize the Nb function and reliability. Libraries of Nbs can be of immune or non-immune source libraries (Tang et al., 2024).

Nb can be displayed on three key platforms: (1) Phage display (fusion of the VHH gene with phage coat proteins such as M13 pIII to express Nbs on the phage surface, forming a selectable library); (2) Yeast display (surface expression of Nbs in Pichia pastoris or Saccharomyces cerevisiae by fusing the VHH gene with yeast cell wall proteins like Aga2, followed by high-affinity clone screening via FACS); and (3) Ribosome display (a cell-free *in vitro* technique that immobilizes mRNA-ribosome-Nb ternary complexes for direct VHH screening without transformation or host cells).

### 4.1 Phage display

Phage display was an efficient technique to produce selective antibodies for tumor-related antigens, viruses, bacteria and other targets. Since being made by McCafferty et al. over 30 years, phage antibody display has become a widely used method for Nb screening (Liu et al., 2021). The construction and validation of camelid-derived nanobody phage display libraries is a standard approach for isolating Nbs against various antigens. Phage display is a method of clonal protein display that inserts the coding DNA for a foreign peptide/protein into the coding gene of phage coat protein gene, thus the exogenous protein is expressed displayed on the phage surface when the phage particle is assembled, and the expressed displayed peptides/proteins can maintain original spatial configuration and biological activity, which can specifically recognize and bind target molecules (Salvador et al., 2019). This approach provides a quick screening process on the large library with much higher efficiency and costs less compared with the conventional hybridomas technique. It displays various scFv libraries on the surface of filamentous phage, selectively binding to the antigen. The *in vitro* evolution is achieved through multiple rounds of biopanning that to enrich the high affinity clones to the corresponding target antigen. Filamentous phage display, lytic phage display and T4 phage display systems are the commonly used phage display systems. Phage display has a broad range of applications, although there are also a number of constraints with regard to its practicality (Jin et al., 2023). Some of them are listed as follows:

#### 4.1.1 Library size and diversity constraints

Phage display requires bacterial transformation and phage packaging, and in some systems, a transmembrane secretion process, which significantly limits the size and molecular diversity of the constructed libraries. Yuan Dong et al. enhanced the scalability of the phage display library by constructing a human scFv phage display library based on the Cre-LoxP recombination system. Advances in biotechnology will enable the development of novel methods to improve the scalability of phage display systems.

#### 4.1.2 Expression bottleneck

There is no guarantee all series can be easily expressed into the phage system because some protein need correct folding, trafficking and insertion into membrane or even complex formation to work. Selective pressure for screening needs to be applied. For instance, disordered folded proteins may be degraded quickly in bacteria cells which requires strenuous control of experimental condition to keep the displayed library intact.

#### 4.1.3 Limited post-construction modifiability

Once a phage display library is constructed, it is difficult to introduce effective *in vitro* mutagenesis or recombination, which restricts the molecular diversity and evolutionary potential of the library.

#### 4.1.4 Toxicity of target molecules

Because phage display is dependent on intracellular gene expression, it is challenging to express and display molecules that are toxic to host cells, such as certain biotoxins.

### 4.2 Bacterial surface display

Bacterial surface display is a technique that utilizes genetic engineering to present functional peptide or protein fragments (target proteins) on the cell surface by fusing them with bacterial outer membrane proteins (carrier proteins). This method relies on membrane-anchoring motifs, which facilitate the translocation of heterologous peptides across the cell membrane to the extracellular surface. Bacterial display offers several advantages, including high transformation efficiency, rapid growth rates, and genetic stability (Duan et al., 2021). In addition, this technique can be combined with flow cytometry for the screening of antibodies specific to target antigens. Fusion of the exogenous protein with the carrier protein can be achieved in three main formats: C-terminal fusion (with the N-terminus anchored in the outer membrane), N-terminal fusion (with the C-terminus anchored), and sandwich fusion (insertion of the foreign protein at a permissive internal site within a carrier protein that contains both a signal peptide and outer membrane anchor sequence). In traditional bacterial surface display systems, the exogenous protein is typically fused to bacterial outer membrane proteins, lipoproteins, or structural subunits of surface appendages (such as pili and flagella). More recently, alternative anchoring scaffolds such as ice nucleation proteins, autotransporters, and S-layer proteins have also been utilized. Early studies primarily focused on Gram-negative bacteria due to their well-characterized genetic backgrounds, which facilitate precise control over protein display. However, from an application standpoint, Gram-positive bacteria offer several advantages: they can tolerate the insertion of larger exogenous proteins, have only a single cytoplasmic membrane to traverse, and possess a thick but accessible cell wall, making them more amenable to surface display (Jovcevska and Muyldermans, 2020).

When combined with fluorescence-activated cell sorting (FACS) or flow cytometric analysis, bacterial surface display becomes a powerful high-throughput screening method. The main advantages of this approach include:

1. The ability to determine the functional properties of each clone within a mutant library and quantitatively analyze enzymatic activity;
2. Currently the only method capable of quantitatively assessing catalytic activity at the single-cell level across large mutant populations;
3. Simultaneous detection of multiple parameters with the capacity to record and track each individual clone.

Compared to phage display, bacterial surface display offers unique advantages in vaccine development, enabling faster and more efficient screening. However, it also presents certain limitations, such as size constraints of displayed proteins, mislocalization, inclusion body formation, and outer membrane instability. Therefore, ongoing efforts are required to develop novel display systems and further optimize the technology for broader applications.

### 4.3 Yeast surface display

Yeast surface display is a eukaryotic protein expression system that has developed rapidly in recent years. The basic principle involves fusing a gene encoding a foreign target protein with the C-terminal coding sequence of a yeast-encoded agglutinin (which contains a GPI anchor signal sequence), and inserting the fusion gene downstream of a signal peptide in a plasmid vector. Upon induction, the fusion protein is directed for extracellular secretion via the signal peptide, and the C-terminal GPI-anchored agglutinin sequence facilitates anchoring of the protein to the yeast cell wall, thereby displaying the protein on the yeast cell surface. This system was developed following the success of phage display technologies. Yeast cells are relatively large, which makes them well-suited for screening and sorting by flow cytometry. Currently, the most commonly used yeast surface display systems are: (1) target protein–α-agglutinin fusions, and (2) α-agglutinin–target protein fusions. In the former, the target protein is fused to the N-terminus of the C-terminal portion of α-agglutinin, and the complex is displayed on the yeast cell surface. The agglutinin is covalently linked to the glucan network of the cell wall through its C-terminal 320 amino acid residues, which are rich in serine/threonine (Ser/Thr) residues. These Ser/Thr-rich regions are extensively O-glycosylated, forming a rod-like structure that acts as a spatial scaffold for display. To date, multiple heterologous proteins have been successfully displayed using the C-terminal domain of α-agglutinin, with the first reported example being α-galactosidase (Muyldermans, 2013).

Currently, yeast surface display has been widely applied in protein–protein interaction studies, directed evolution, and novel vaccine development. In terms of directed evolution, this system has been successfully used to evolve proteins for improved affinity and stability, with one of the earliest applications being antibody affinity maturation (McMahon et al., 2018). In vaccine development, yeast cells can serve as live oral vaccines. Proteins displayed on the yeast surface are readily accessible to antibodies and thus easily recognized by the immune system. Even small peptides can become immunogenic when displayed on the cell surface, making yeast a promising platform for expressing heterologous antigenic proteins for vaccine development. Yeast surface display offers several advantages. It mimics the natural process of antibody selection that occurs in the human immune system and enables the identification of polyclonal populations with different binding specificities in a single experiment (Kajiwara et al., 2020). Compared to phage and bacterial display systems, yeast enables post-translational modifications and correct folding of complex eukaryotic proteins. Moreover, glycosylation improves antibody solubility, and co-expression of chaperone proteins facilitates proper folding in the endoplasmic reticulum. Although the library capacity is relatively limited (~10^7^–10^9^ clones), the diversity is high, and unique high-affinity antibodies can be readily isolated with simple screening steps.

## 5 The application of Nbs

Nbs have shown broad application prospects in multiple fields such as biomedicine, diagnosis, treatment and biotechnology due to their unique structural advantages, stability and good affinity. The following is an overview of its application value from several main directions:

### 5.1 Application in inhibition and monitoring of virus infection

PRRSV (Porcine Reproductive and Respiratory Syndrome Virus) is a significant swine pathogen. As of now, it is lack of broadly applicable anti-PRRSV strategies. Recently, Deng et al. identified several high affinity anti-CD163 Nbs. Of interest, Nb2 exhibited substantial inhibitory effects on diverse PRRSV lineages and suppressed virus-related NF-κB signaling by interfering with viral attachment and reducing CD163 transcription. In particular, the SRCR5 domain of CD163, a critical region implicated in PRRSV infection, was recognized by these Nbs, thereby facilitating the establishment of broad-spectrum approaches against PRRSV. Similarly, Yang et al. developed Nb-peptide conjugates (NPCs) by fusing PRRSV-specific, non-neutralizing Nbs with peptides derived from CD163 that recognize the receptor-binding domain (RBD) of PRRSV proteins. These NPCs showed potent inhibitory activities against diverse lineages of PRRSV (Yang H. et al., 2024). In addition, Zhou et al. screen a nanobody with the neutralizing activities for PRRSV. By integrating the adhesion protein and Nb, the probiotics enhanced its adhesion to IPEC-J2 cells and thus was utilized to prevent PRRSV fecal transmission (Zhou et al., 2024).

Notably, Nbs were also employed to develop enzyme-linked immunosorbent assay for PRRSV, indicating high sensitivity and accuracy (Duan et al., 2021; Sun et al., 2024). Importantly, the Nb could suppress PRRSV replication by inhibiting self-interaction of the viral nucleocapsid protein (Duan et al., 2024).

African swine fever virus (ASFV) is a virulent swine pathogen that causes huge losses to the swine industry. ASFV is large in size, thus vaccines of great efficacy are in urgent needed; however, Nbs targeting ASFV proteins are developed by many research groups of monitoring. Nbs of CD2v, p54, and p72 have been used to develop ELISA kit to monitor ASFV infection (Zhang et al., 2022; Zhao H. et al., 2022; Zhao J. et al., 2022; Zhu et al., 2024). Interestingly, Nbs are also helpful to identify epitopes of host immnuno-recognization. Zhao et al. screened one Nb via utilizing ASFV P54. The epitope recognized by this Nb also was reactive to the inactivated serum of ASFV naturally infected pigs (Zhao et al., 2023). Similarly, Wei et al. identified an epitope by utilizing ASFV K205R under the Nb verification (Wei et al., 2025). Notably, the structural proteins of ASFV, including p30, p54, and p72, were utilized to generate Nb-based TRIM-aways. These constructs exhibited significant potency in degrading viral structural proteins, thereby underscoring potential pathways for the development of novel antiviral strategies against ASF (Yang F. et al., 2024).

Highly pathogenic avian influenza viruses present significant threats to the poultry industry, public health, and have the potential to induce global pandemic risks. Nbs have extensive applications in the detection, differential diagnosis and prevention of avian influenza viruses. For example, Xu et al. developed a Nb of broadly neutralizing versatile clades of IAV H5 subtype by binding to HA1 (Xu et al., 2025). Chen et al. isolated a Nb with cross-group neutralization across various IAV subtypes. Critically, the epitope recognized by this Nb could elicits cross-reactive antibodies and provides partial protection by lethal viral challenges (Chen et al., 2025). In addition, Ji et al. established a Nb-based competitive ELISA (cELISA) in monitoring anti-IAV antibodies (Ji et al., 2022). Interestingly, a Nb-based reporter system was used for living cell sensing of IAV infection, providing a robust and advanced tool for the analysis of IAV infections.

Notably, besides interfering with viral infection, the Nb was also utilized as the cellular delivery vehicle. For instance, the FMDV particles were delivered by Nbs into dendritic cells to enhance host immune responses (Cheng et al., 2023).

### 5.2 Application in inhibition and monitoring of bacteria infection

*Staphylococcus aureus* (*S. aureus*) is capable of producing a variety of toxins, including staphylococcal enterotoxin type B and C (SEB/C), which play a critical pathogenic role in host infections and foodborne intoxications. As described above, Nbs were also employed to detect SEB/C (Graef et al., 2011; Ming et al., 2023) and clumping factor A (ClfA) (Mei et al., 2024). Nbs were also proved to neutralize SEB and thus hold promises for combating SEB-relative diseases. Detailed information demonstrated Nbs bound to SEB at the T-cell receptor interface, blocking the SEB toxicity (Zong et al., 2024). Zhang et al. develop a Nb (plus RNAbody)-based sandwich ELISA system, which could readily detect S. aureus α-hemolysin in milk and pork (Zhang et al., 2024).

*Bacillus anthracis* (*B. anthracis*) is a zoonotic spore-forming pathogen, can lead to anthrax, which is a highly resilient and deadly for animals and humans. It can secret toxins into the bloodstream once the infection occurred. Cecil et al. characterized a group of Nbs against the anthrax toxins via binding to the *B. anthracis* edema factor and lethal factor, which thus prevented entry of the toxin into the cells. Interestingly, Nbs with neutralizing function protected mice against the lethal anthrax spore infection, indicating a potent blocking role in *in vivo* tests (Vrentas et al., 2016).

Nbs inhibited the self-assembly of the S-layer proteins Sap of *B. anthracis* attenuated its growth and pathology *in vivo*. Subcutaneous delivery of these inhibitory Nbs cleared *B. anthracis* infection and prevented mice lethality, highlighting a therapeutic intervention of *B. anthracis* infection (Fioravanti et al., 2019). Recent work established a system of molecular dynamics simulations of these inhibitory Nbs, paving a road for Nb therapeutics (Cecil et al., 2024).

In addition, Nbs targeting the protective antigen of anthrax toxin were also developed. Nbs fused with the β-gal targeted the Bacillus collagen-like protein of anthracis (BclA) enabled sensitive detection of *B. anthracis*.*Mycobacterium tuberculosis* (*M. tuberculosis*), the etiological agent responsible for tuberculosis, exhibits high pathogenicity and results in severe morbidity and mortality.

Recently, Fay et al. developed a system by using the anti-ALFA Nb fused with a fluorescent protein to recapitulate the protein localization by fluorescent microscopy in living cells, serving as a versatile platform for the discovery of protein biology in mycobacteria (Fay et al., 2025). It was found that the Nb specific for ESAT-6 could inhibit *M. tuberculosis* growth in macrophages (Bates et al., 2024). Nb was also utilized to stabilize the *M. tuberculosis* to dissect the cryo-EM structure of its adenylyl cyclase transmembrane region (Mehta et al., 2022). Nbs were also applied to detection of *Escherichia coli* (*E. coli*) in food samples (He et al., 2025). They were also generated to bind to the extracellular fimbriae of enterotoxigenic *E. coli* (ETEC)/Shiga toxin producing *E. coli* (STEC) and thus blocked their adhesion in the gastrointestinal tract (Moonens et al., 2014).

It should be noted that *E. coli* and *Lactobacillus* could be utilized as delivery vectors to display the Nbs. Many researches applied these systems to produce and purify Nbs. For example, the E. coli could be utilized as a tool to screen Nbs capable of binding to and neutralizing severe acute respiratory syndrome coronavirus 2 (SARS-CoV-2) and bovine viral diarrhea virus (BVDV) (Liu et al., 2024; Ma et al., 2024; Zhao et al., 2025).

Oral administration of Nbs was utilized to prevent GI *E.coli* infection. *Lactococcus lactis* was used to deliver Nbs to inhibit norovirus infection (Yuki et al., 2022).

### 5.3 Application in inhibition and monitoring of parasite infection

In addition to their applications in viruses and bacteria research, Nbs were also used to inhibit parasite infection. For example, the Nbs could suppress the trypanosomatid pyruvate kinases activity by an allosteric mechanism (Pinto Torres et al., 2025). Trypanosoma brucei is a parasitic protozoan causing African trypanosomiasis, or sleeping sickness, transmitted by infected tsetse flies to animals and humans. Neutralization of Trypanosoma brucei Q586B2 Nbs hampered myeloid-derived IL-10 production and lowered parasitemia (Stijlemans et al., 2024). Genetically engineered Sodalis glossinidius expressing Nbs significantly compromised Trypanosoma brucei development in the tsetse fly midgut (De Vooght et al., 2022). Interestingly, Broster Reix et al. demonstrated Nbs also were able to suppress Trypanosoma brucei propagation. They screened one Nb targeting the T. brucei cytoskeletal protein Tb BILBO1, which could killed parasites producing phenotypes similar to the RNA knockdown, suggesting Nbs should be used to prevent T. brucei infection (Broster Reix et al., 2021). Notably, because Nbs do not contain the Fc fragments, they should hold less background interference in the ELISA assay. Another commonly causing agent of African trypanosomiasis is Trypanosoma congolense, which continues to impose a heav burden in livestock of Sub-Saharan Africa. Nbs were employed to establish the ELISA assay and improved Nb-based lateral flow assay to monitor Trypanosoma congolense infection, which showed increased sensitivity in detection of cattle samples (Odongo et al., 2016; Pinto Torres et al., 2018). Similarly, Abeijon et al. established an ELISA assay using Nbs specific for visceral leishmaniasis-an important zoonotic parasite, which was beneficial to increase the kit sensitivity (Abeijon et al., 2018).

Toxoplasma gondii (T. gondii) is a critical zoonotic pathogen which can infect a broad range of hosts, including cats, dogs and humans. Pathogenic mechanism study could help uncover the cellular processes of T. gondii within the hosts. By employing the actin Nbs, Periz et al. revealed how the dynamic F-actin networks connected Toxoplasma progeny and spread in the replicative vacuole (Periz et al., 2017), which should provide hints for novel strategies to decline intracellular T. gondii infection.

## 6 Summary and outlook

Although conventional monoclonal antibodies remain the primary molecular tools in biomedical research, disease diagnosis, and therapy, their high production cost, poor stability, large molecular size, and limited tissue penetration restrict their applications in certain fields. Nbs, as a novel class of antibody fragments, overcome these limitations by exhibiting small molecular size, excellent thermal stability, high expression efficiency, strong tissue penetration, and the ability to recognize hidden epitopes (Muyldermans, 2013). Consequently, Nbs have attracted considerable attention in cancer, neurological disorders, autoimmune diseases, infectious diseases such as HIV, toxin-related diseases, and fundamental research. In the field of animal infectious diseases, researchers worldwide have targeted whole pathogens, viral structural and non-structural proteins, and viral replication enzymes to screen a variety of Nbs using phage display, yeast display, and bacterial display technologies. These efforts have significantly advanced the development of diagnostic and therapeutic tools for animal disease prevention and control (Salvador et al., 2019). Notably, advances in artificial intelligence enable more precise identification of Nbs with potential pathogen-binding affinity, significantly accelerating their development in clinical applications.

Conventional antibody-based kits in the diagnostics, however, are fragile with poor thermal stability, require the cold-chain logistics, high detection price, and not fully specific/sensitive that does not support early/differential diagnosis. The high affinity and great thermal stability of Nbs allows the preparation of low cost but high specificity/sensitivity and room temperature stable diagnostic reagents for easy detection at the field. In therapy, pathogens such as foot-and-mouth disease virus (FMDV) and influenza A virus (IAV) exhibit multiple serotypes and high antigenic variability, posing challenges for vaccine-induced immunity. Due to their small size and elongated CDR3 loops, Nbs can recognize conserved and functionally critical hidden epitopes located within protein conformational clefts that are inaccessible to conventional antibodies, making Nbs ideal candidates for broad-spectrum neutralizing agents. Moreover, Nbs demonstrate remarkable resistance to proteolytic enzymes like pepsin and trypsin and tolerate harsh physicochemical conditions, allowing administration via inhalation or oral routes at large doses, which is advantageous for mass treatment in livestock and poultry, especially for respiratory and gastrointestinal diseases such as respiratory syncytial virus (RSV) and porcine epidemic diarrhea virus (PEDV).

As a promising intracellular antibody format, Nbs have shown preliminary success in antiviral applications inside cells. Future efforts may focus on screening Nbs against intracellular pathogens such as Brucella spp., utilizing intracellular Nb delivery to control such infections. However, safe and efficient intracellular delivery remains a major bottleneck; viral vector-based delivery systems, such as adenovirus vectors, offer promising solutions for large-scale intracellular Nb application.

1. Despite these advances, several challenges remain in the practical use of Nbs:
2. How to obtain Nbs with even higher affinity and neutralization potency;
3. How to enhance Nb cell membrane penetration for intracellular targeting;
4. How to prolong Nb half-life *in vivo* to improve therapeutic durability;
5. How to address infections where cellular immunity plays a predominant antiviral role;
6. How to establish efficient screening platforms for broad-spectrum neutralizing Nbs;
7. How to reduce Nb immunogenicity across different animal species.

Strategies such as engineering multivalent, multispecific, or biparatopic Nbs, and fusing Nbs with Fc fragments or cell-penetrating peptides (CPPs), provide new avenues to tackle these challenges. With continued improvements in screening techniques, expression systems, and protein engineering, Nbs are poised to play a pivotal role in animal disease prevention and control, with their applications in diagnostics, therapeutics, and research expected to expand significantly in the near future.
